# The Chitinolytic Activities of *Streptomyces* sp. TH-11

**DOI:** 10.3390/ijms12010056

**Published:** 2010-12-27

**Authors:** Kim-Chi Hoang, Tzu-Hsuan Lai, Chung-Sheng Lin, Ying-Tsong Chen, Chun-Yi Liau

**Affiliations:** 1 General Education Center, Ta Hwa Institute of Technology, HsinChu 307, Taiwan; E-Mail: chi@thit.edu.tw; 2 Departments of Bioengineering, Tatung University, Taipei 104, Taiwan; E-Mail: sonic_9001@hotmail.com; 3 School of Medicine, Chung Shan Medical University and Department of Internal Medicine, Chung Shan Medical University Hospital, TaiChung City 402, Taiwan; E-Mail: lcs@csmu.edu.tw; 4 Institute of Genomics and Bioinformatics, National Chung Hsin University, TaiChung City 402, Taiwan; E-Mail: onion@nchu.edu.tw; 5 Division of Molecular and Genomics Medicine, National Health Research Institutes, Miaoli 350, Taiwan

**Keywords:** chitin, Streptomyces, chitinase

## Abstract

Chitin is an abundant biopolymer composed of units of *N-*acetyl-D-glucosamine linked by β-1,4 glycosidic bonds. Chitin is the main component of the shells of mollusks, the cell wall of fungi and yeast and of the exoskeleton of crustaceans and insects. The degradation of chitin is catalyzed by chitinases that occur in a wide range of organisms. Among them, the chitinases from microorganisms are extremely important for the degradation and recycling of the carbon and nitrogen trapped in the large amount of insoluble chitin in nature. *Streptomyces* sp. TH-11 was isolated from the sediment of the Tou-Chien River, Taiwan. The chitinolytic enzyme activities were detected using a rapid in-gel detection method from the cell-free preparation of the culture medium of TH-11. The chitinolytic enzyme activity during prolonged liquid culturing was also analyzed by direct measurement of the chitin consumption. Decomposition of the exoskeleton of shrimps was demonstrated using electron microscopy and atomic force microscopy.

## 1. Introduction

Chitin is a linear polysaccharide made of β-1,4 linked *N*-acetyl-D-glucosamine (GlcNAc). More than 1 × 10^10^ to 1 × 10^12^ tones of chitin are produced annually in terrestrial and marine habitats, making it the second most abundant polysaccharide in nature, after cellulose. Chitin is insoluble in water and exists with different degree of acetylation, different molecular sizes (up to several MDa), and can associate with other organic and inorganic compounds. It occurs mainly as a structural component in the cell walls of fungi and yeast and the exoskeletons of insects and crustaceans. Chitosans are components derived by enzymatic deacetylation of chitin and are found in the cell walls of certain fungi [1].

In a previous study, we reported a simple and rapid detection technique for in-gel glycol chitosan activity staining [2]. The samples containing chitinolytic enzymes were prepared with β-mercaptoethanol-free loading buffer and separated by electrophoresis utilizing 12% SDS–PAGE containing 0.01% (w/v) glycol chitosan. Clearance of the embedded glycol chitosan by the enzymes can be detected after electrophoresis by a modified Coomassie Brilliant Blue G 250 staining [3].

Streptomycetes are soil-dwelling mycelial bacteria that produce a large number of secreted proteins and many secondary metabolites, including important antibiotics. Chitin is a major nutrient source for many streptomycetes, and these microorganisms have developed complex extracellular systems for chitin utilization [4]. Analyses of the genomes of various *Streptomyces* strains have revealed many chitinase genes, potentially enabling them to hydrolyze the diverse chitin types they encounter. For example, analysis of the genomic sequence of a *Streptomyces coelicolor* strain revealed as many as 13 putative chitinases [5,6]. Nowadays, chitinases have been purified from various *Streptomyces* strains, but detailed studies on the enzymes that degrade chitin form exoskeletons or fungal walls are, however, scarce.

*Streptomyces* sp. TH-11 was isolated from the sediment of the Tou-Chien River in Taiwan in a previous study [7]. A total of 305 isolates from different regions of the river were screened in the study. Twelve strains were found to be capable of degrading poly(β-hydroxybutyrate) (PHB), poly(ɛ-caprolactone) (PCL) and poly(ethylene succinate) (PES) and TH-11 is one of the best degraders [7,8]. According to 16S rRNA sequence comparison, TH-11 shows 99% sequence identity with *Streptomyces viridochromogenes*, a strain that produces several chitinolytic enzymes. Since chitin is abundant in the natural habitat of TH-11, the chitinolytic activities of this strain were analyzed using chitin powder, glycol chitosan and raw shrimp shells in the present study.

## 2. Results and Discussion

### 2.1. Degradation of Chitin Powder by *Streptomyces* sp. TH-11

**Figure 1:**
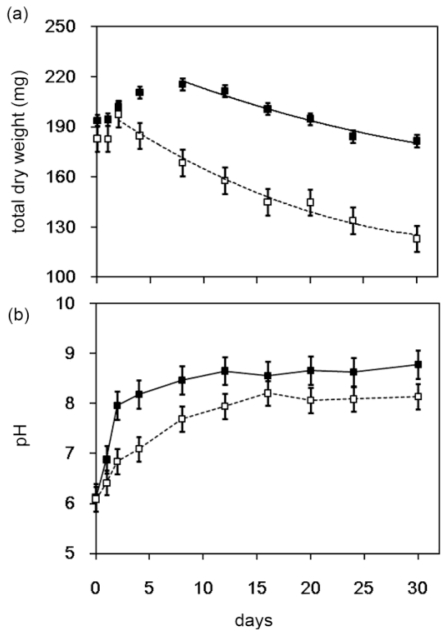
(**a**) Chitin powder degradation test (30 days). The dry-weight (mg) of the culture was measured after inoculation. Decrease of the total dry weight for samples with (solid square) or without (open square) gelatin (1 mg/mL); (**b**) Change of pH value of the culture media during the chitin powder degradation test. Samples cultured with or without gelatin (1 mg/mL) are shown by solid squares and open squares, respectively.

*Streptomyces* sp. TH-11 was cultured with degradation media containing 0.1% (w/v) chitin powder (Wako Pure Chem., Japan) with vigor agitation at 30 °C. The total dry weight of the culture was measured over 30 days (Figure 1a). Since many of the hydrolytic activities such as esterase or PES depolymerase of *Streptomyces* were reported to be inducible by addition of proteins such as gelatin to the culture media, the effect of gelatin addition was also measured. The degradation of chitin powder, demonstrated by the decline of total dry weight, was not enhanced by gelatin addition (Figure 1a). The cell growth, which is enhanced by gelatin addition, may have contributed to the increase of total dry weight at the early stage of culturing. It has been shown in a PES depolymerase study that prolonged culturing resulted in an increase of pH [8]. In the present chitin powder degradation test, a similar increasing of pH value of the culture media is also observed during prolonged culturing (Figure 1b). Specific chitinolytic activity (U/mg total protein) in the medium produced by *Streptomyces* sp. TH-11 during the 30 days of the chitin powder degradation test was also tested after removal of cells and insoluble material by centrifugation and filtration. The highest specific chitinolytic activity (~1100 U/mg total protein) was detected at day 12 to 16. It had also been reported that up to eight days of incubation is necessary for *Streptomyces* strains to reach maximum chitinase activity production in optimized liquid culture conditions [9].

### 2.2. In-gel Activity Staining of Chitinolytic Enzymes from *Streptomyces* sp. TH-11

**Figure 2:**
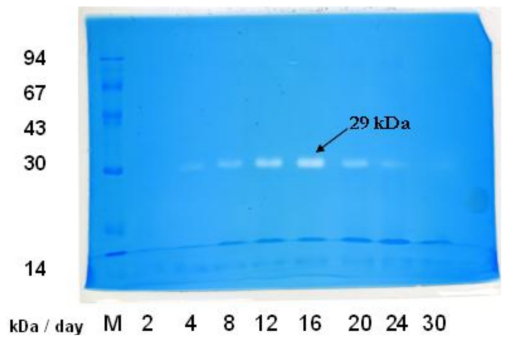
Detection of the chitinolytic activity in the culture medium of TH-11 during the chitin-powder degradation test. The cell-free samples prepared from the culture media were separated in a glycol chitosan-embedded SDS-PAGE and then stained using a previously reported method [3]. The 29 kDa clear zones, which appeared to be most obvious at day 16, are indicated.

Culture media from the chitin powder degradation experiments were also collected by removal of the cells and other insoluble substances, and subjected to in-gel chitinolytic activity staining using a previously published method [3]. The degradation of embedded glycol chitosan (Sigma-Aldrich, MO, U.S.) resulted in clear zones visualized by Coomassie Brilliant Blue G 250 (Figure 2). A clear zone corresponding to a MW of about 29 kDa was observed and appeared to be expressed in highest yield at day 12–16. This is consistent to the specific chitinolytic activity of the culture medium measured by reducing end production, which is also highest at day 12–16.

Chitinases of various molecular weights have been identified in various *Streptomyces* strains [10]. These including a 20 kDa chitinase from *Streptomyces* sp. M-20, 28 kDa, 25 kDa, and 45 kDa from *Streptomyces* sp. NK 1057, 45 kDa and 45 kDa from *S. albovinaceus* S-22, 45 kDa from *Streptomyces* sp. ANU 6277, and 49 kDa from *S. griseus* HUT 6037 [11,12]. Based on sequence similarity, chitinases are classified into two families: 18 and 19. Chitinases belong to family 18 are widespread among a variety of organisms, but those of family 19 are found almost only in higher plants. For *Actinobacteria*, the distribution of family 19 chitinase was systematically studied, and it was suggested that that family 19 chitinases of *Streptomyces* species were acquired from plants by horizontal gene transfer [13]. Genomics analyses on *Streptomyces coelicolor* A3(2) genome have identified 11 family 18 and two family 19 chitinases [6]. Among these, a 29 kDa family 19 chitinase Chi19F was identified with high activity toward glycol chitin and soluble chitin [6]. Identification of the glycol chitosan hydrolyzing activity at 29 kDa in our study makes an addition to this repertoire. Further enzymatic studies are, however, necessary to characterize the 29 kDa chitinase.

### 2.3. Decomposition of Shrimp Shells by *Streptomyces* sp. TH-11

**Figure 3:**
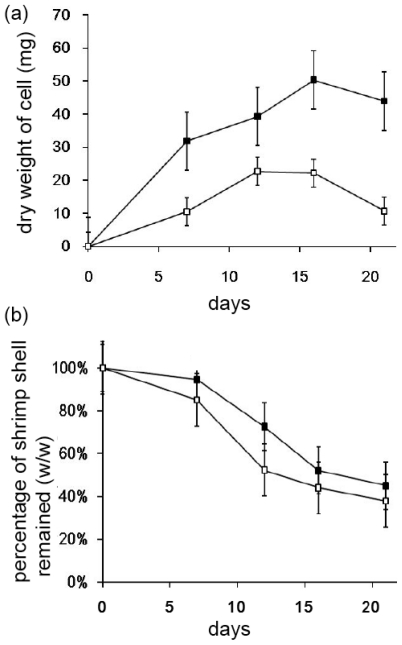
(**a**) Growth of *Streptomyces* sp. TH-11 on media containing ground shrimp shells; (**b**) Dissipation of shrimp shells monitored by direct weight measurement. Samples cultured with or without gelatin (1 mg/mL) are shown by solid squares and open squares, respectively.

It is estimated that chitin composes 20% of raw shrimp shells in weight [14]. To test the degradation of shrimp shells by *Streptomyces* sp. TH-11, the bacteria were cultured with degradation media containing 0.1% (w/v) dry and grinded shrimp shells with vigorous agitation at 30 °C. The total dry weight of the bacteria in the culture medium was measured over 21 days (Figure 3a). The shrimp shells were removed from the medium and the dry weights were measured to monitor the dissipation (Figure 3b). In 20 days, about half of the insoluble shrimp shells were diminished. Addition of 1 mg/mL gelatin to the media did not enhance the dissipation of shrimp shells by TH-11, but supported better cell growth.

### 2.4. Decomposition of Shrimp Shells Visualized by SEM and AFM

**Figure 4:**
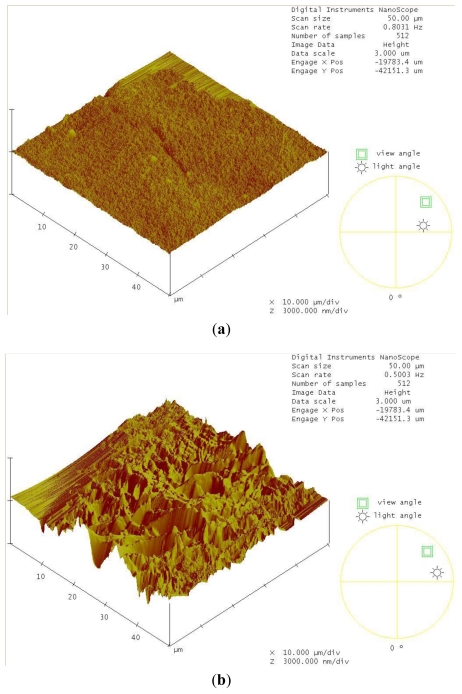

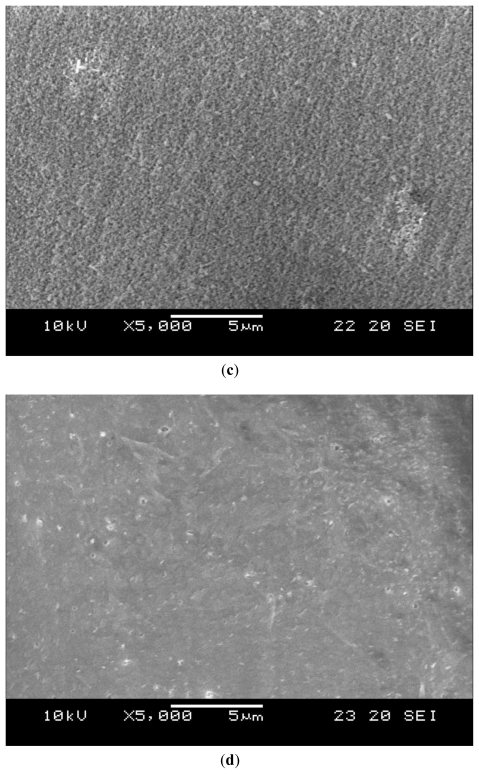

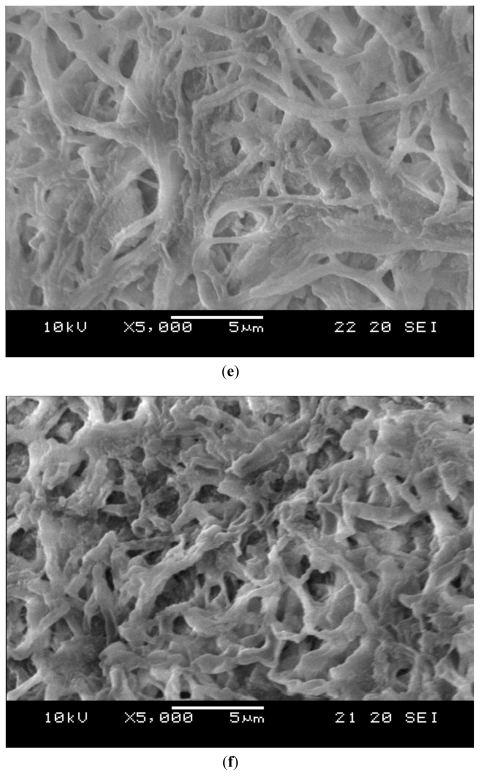
(**a**) The shrimp shells at day 0 visualized by atomic force microscopy (AFM); (**b**) The shrimp shells after 7 days of incubation with TH-11 visualized by AFM; (**c**) Scanning electron microscopy (SEM) of the shrimp shells at day 0; (**d**) SEM on the shrimp shells at day 7; (**e**) SEM on the shrimp shells at day 12; (**f**) SEM on the shrimp shells at day 16. Panels (c)–(f): magnification is 5000 ×.

Atomic force microscopy (AFM) was utilized to detect the changes on the surface structure of the shrimp shells in the decomposition experiment. A comparison between day 0 and day 7 is shown (Figure 4a, b). The decomposition of shrimp shells was also demonstrated by scanning electron microscopy (SEM) at day 0, day 7, day 12 and day 16 (Figure 4c, d, e, f). Compared with the original surface at day 0, break down of the shrimp shell is apparent after incubation with *Streptomyces* sp. TH-11. *Streptomycetes* contain many chitinase genes to hydrolyze diverse chitin types with different molecular sizes, degree of acetylation, and associated organic/inorganic compounds. The aforementioned chitinolytic activity detected at 29 kDa, presumably a family 19 chitinase that is active on a soluble substrate, may not be the enzyme responsible for the decomposition of shrimp shells since family 19 chitinases are not active toward crystalline substrates [6]. On the other hand, some of the chitinases may not have sufficient activity toward glycol chitosan and may not appear in the zymogram shown in Figure 2.

## 3. Experimental Section

*Streptomyces* sp. TH-11 was isolated from soil samples collected from the sediment of Tou-Chien River, Taiwan [7]. *Streptomyces* was cultured in modified Starch-Casein medium (Starch 4 g, Casein 0.12 g, KNO_3_ 1.6 g, NaCl 1.6 g, K_2_HPO_4_ 1.6 g, MgSO_4_·7H_2_O 40 mg, CaCO_3_ 16 mg, FeSO_4_·7H_2_O 8 mg in 1000 mL water, pH 7) containing 50 μg/mL cycloheximide. Separation and detection of chitinolytic enzymes in glycol chitosan-containing polyacrylamide gel by electrophoresis and modified Coomassie Brilliant Blue G 250 staining were performed as previously described [3]. Degradation media was prepared by mixing 1 g of chitin powder (Wako Pure Chem., Japan) in 1 L medium containing 0.1 g yeast extract, 10 mg FeSO_4_·7 H_2_O, 0.2 g MgSO_4_·7 H_2_O, 1 g (NH_4_)_2_SO_4_, 20 mg CaCl_2_·2 H_2_O, 0.1 g NaCl, 0.5 mg Na_2_MoO_4_·2 H_2_O, 0.5 mg Na_2_WO_4_·2 H_2_O, 0.6 mg MnSO_4_·H_2_O, and detergent (CH_3_(CH_2_)_n_COONa, *n* = 6~14, Nice Co., Taiwan). Weight of the cells and remaining chitin in the liquid media after prolonged incubation were performed by direct measurement of the dry weight.

Chitinolytic activity was determined by measuring the reducing end group produced from glycol chitosan (Sigma-Aldrich, MO, U.S). The reaction mixture, consisting of 100 μL of enzyme solution, 36 μL of 25 mg/mL glycol chitosan (a final concentration of 1 mg/mL) and 764 μL of 0.1 M sodium acetate buffer, pH 5.0, was incubated at 37 °C for 30 min, and then the reaction was terminated by heating in boiling water for 15 min. The reducing end group produced was measured colorimetrically by potassium ferricyanide assay [15]: the reaction solution was mixed with 1.0 mL potassium ferricyanide reagent, heated in boiling water for 15 min, and then subjected to spectrophotometry measurement at 410 nm. One unit of chitinase activity was defined as capable of releasing reducing ends corresponding to 1 μg of GlcNAc from glycol chitosan in an hour. Pacific white shrimp (*Litopenaeus vannamei*) was purchased from a local market. After boiling in water, the shrimp shell was peeled off, sun-dried and crushed.

Atomic force microscopy was performed using Dimension 3100 AFM with NS3a controller (Digital Instruments/Veeco Inc., Santa Barbara, CA, U.S.). Scanning electron microscopy was performed using a Hitachi S-570 (Hitachi, Japan).

## 4. Conclusions

Chitinolytic systems of *Streptomyces* are usually composed of a diversity of enzymes with different specificities. In this study, chitinolytic activities of *Streptomyces* sp. TH-11 were demonstrated using three different substrates: chitin powder, glycol chitosan, and shrimp shells. Activity staining on the cell-free culture medium indicated an activity zone on embedded glycol chitosan at an approximate molecular weight of 29 kDa. The decomposition effects of shrimp shells by TH-11 were apparent as visualized using SEM and AFM, in addition to the reduction in shrimp shell mass. Taken together, as one of the best degraders we screened from the river sediment with depolymerase activities, the strain is also capable of decomposing different chitin substrates including β-chitin (crystalline powder), deacetylated chitin (glycol chitosan), and raw shrimp shells, presumably by producing different chitinolytic enzymes.

## References

[1] Kumar MN Muzzarelli RA Muzzarelli C Sashiwa H Domb AJ Chitosan chemistry and pharmaceutical perspectives Chem. Rev 2004 104 6017 6084 15584695 10.1021/cr030441b

[2] Liau CY Lin CS Detection of chitinolytic enzymes in *Ipomoea batatas* leaf extract by activity staining after gel electrophoresis J. Chin. Chem. Soc 2008 55 678 681

[3] Liau CY Lin CS A modified Coomassie Brilliant Blue G 250 staining method for the detection of chitinase activity and molecular weight after polyacrylamide gel electrophoresis J. Biosci. Bioeng 2008 106 111 113 18691542 10.1263/jbb.106.111

[4] Chater KF Biro S Lee KJ Palmer T Schrempf H The complex extracellular biology of *Streptomyces* FEMS Microbiol. Rev 2009 34 171 198 20088961 10.1111/j.1574-6976.2009.00206.x

[5] Bentley SD Chater KF Cerdeño-Tárraga AM Challis GL Thomson NR James KD Harris DE Quail MA Kieser H Harper D Complete genome sequence of the model actinomycete *Streptomyces coelicolor* A3(2) Nature 2002 417 141 147 12000953 10.1038/417141a

[6] Kawase T Yokokawa S Saito A Fujii T Nikaidou N Miyashita K Watanabe T Comparison of Enzymatic and Antifungal Properties between Family 18 and 19 Chitinases from *S. coelicolor* A3(2) Biosci. Biotechnol. Biochem 2006 70 988 998 16636468 10.1271/bbb.70.988

[7] Hoang KC Lee CY Tseng M Chu WC Polyester-degrading actinomycetes isolated from the Touchien River of Taiwan World J. Microbiol. Biotechnol 2007 23 201 205

[8] Hoang KC Lee CY Lai YC Liau CY TH-11, a *Streptomyces* sp. strain that degrades poly(3-hydroxybutyrate) and poly(ethylene succinate) J. Chin. Chem. Soc 2008 55 1214 1220

[9] Nawani NN Kapadnis BP Optimization of chitinase production using statistics based experimental designs Process Biochem 2005 40 651 660

[10] Hjort K Bergstrom M Adesina MF Jansson JK Smalla K Sjöling S Chitinase genes revealed and compared in bacterial isolates, DNA extracts and a metagenomic library from a phytopathogen-suppressive soil FEMS Microbiol. Ecol 2010 71 197 207 19922433 10.1111/j.1574-6941.2009.00801.x

[11] Bhattacharya D Nagpure A Gupta RK Bacterial chitinases: properties and potential Critical Rev. Biotechnol 2007 27 21 28 17364687 10.1080/07388550601168223

[12] Narayana KJP Vijayalakshmi M Chitinase Production by Streptomyces sp. ANU 6277 Braz. J. Microbiol 2009 40 725 733 10.1590/S1517-83822009000400002 PMC3768568 24031419

[13] Kawase T Saito A Sato T Kanai R Fujii T Nikaidou N Miyashita K Watanabe T Distribution and Phylogenetic Analysis of Family 19 Chitinases in *Actinobacteria* Appl. Environ. Micorbiol 2004 70 1135 1144 10.1128/AEM.70.2.1135-1144.2004 PMC348904 14766598

[14] Percot A Viton C Domard A Optimization of chitin extraction from shrimp shells Biomacromolecules 2003 4 12 18 12523840 10.1021/bm025602k

[15] Imoto T Yagishita K A simple activity measurement of lysozyme Agric. Biol. Chem 1971 35 1154 1156

